# Acute stimulation of mesenchymal stem cells with cigarette smoke extract affects their migration, differentiation, and paracrine potential

**DOI:** 10.1038/srep22957

**Published:** 2016-03-15

**Authors:** Elizabeth A. Wahl, Thilo L. Schenck, Hans-Günther Machens, J. Tomás Egaña

**Affiliations:** 1 Department of Plastic Surgery and Hand Surgery, University Hospital rechts der Isar, Technische Universität München, Munich, Germany; 2 Institute for Medical and Biological Engineering, Schools of Engineering, Biological Sciences and Medicine, Universidad Católica de Chile, Santiago, Chile

## Abstract

Mesenchymal stem cells (MSCs) are known to play a key role in tissue regeneration, while smoking cigarettes is described to impair it. This work focuses on the effect cigarette smoke extract (CSE) has on the migration, differentiation, and paracrine potential of human adipose derived MSCs (AdMSCs). To mimic native conditions *in vitro,* AdMSCs were cultured in either monolayer or three-dimensional pellet cultures. While constant exposure to high concentrations of CSE had lethal effects on AdMSCs, lower concentrations of CSE impaired cell migration when compared to control conditions. The secretion of key interleukins was downregulated when CSE was exposed to the cells at low concentrations. Moreover, in this work AdMSCs were exposed to CSE while simultaneously being induced to differentiate into adipocytes, osteoblasts, and chondrocytes to determine the effect of CSE on the cells potential to differentiate. While adipogenic differentiation showed no significant variation, AdMSCs exposed to osteogenic and chondrogenic supplements showed both early and late genetic level variation when acutely exposed to low concentrations of CSE. Our results indicate that even a small amount of cigarette smoke can have detrimental effects on the regenerative potential of MSCs.

Cigarette smoking is a widespread and major clinical issue, despite governmental and societal approaches to limit smoking, such as higher taxation and smoking bans. Cigarette smoke (CS) consists of a complex mixture of chemicals that contain as many as 7,000 compounds, of which 250 are known to be toxic and can quickly be absorbed into the bloodstream^1^. The toxic effects of CS undermine the conditions that are required for proper tissue repair^2^, as well as significantly increase the risk of cardiovascular disease, rheumatoid arthritis, osteoporosis, and cancer^3^,^4^,^5^.

CS results in slower cell division, induces DNA damage, inhibits healing time, and tissue repair capabilities by reducing nutritional blood flow^2^,^6^,^7^. Furthermore, CS has shown to cause irreversible arterial damage^8^. Nicotine, the chief component in tobacco, has been widely studied as a sole toxic ingredient, It has been show to inhibit the function of red blood cells causing significant vasoconstriction and decreased levels of oxygen in the blood^9^. Moreover, nicotine is associated with increased platelet aggregation, decreased prostacyclin formation, as well as increased blood viscosity, which can result in microvascular thrombosis^10^,^11^,^12^,^13^. Fibroblasts and macrophages, constituents responsible for clotting and healthy scarring of tissue, are also reduced in the exposure of nicotine. Further toxic components such as nitric oxide, hydrogen cyanide, and tar also have detrimental effects^14^,^15^. Slower healing was reported in smokers with wounds resulting from disease, trauma, and surgical procedures leading to increased mortality and morbidity rates, as well as extended hospital stays^6^,^16^. Furthermore, tissue necrosis risk has been shown to increase linearly by pack consumption^17^.

Mesenchymal stem cells (MSCs) are key to tissue regeneration and repair^18^, they are able to synthesize a wide range of cytokines and growth factors that have profound effects on local cellular dynamics. These cells can act as trophic mediators, releasing pro-angiogenic, chemotactic, and immune modulatory factors, which play an important role in wound healing, bone and cartilage remodelling, and overall tissue regeneration^19^,^20^.

The work presented here examines the effects of cigarette smoke extract (CSE) as a whole and addresses the question of how it affects the regenerative potential of adipose derived MSCs (AdMSCs) via their migration, differentiation by means of genetic expression, and paracrine potential. We analysed the migration and cell viability under different concentrations of CSE to test the effects of the oxidizing agents and radicals in CS^21^. Moreover, we examined the damaging effects of CS on the release of bioactive factors from MSCs, which could in turn compromise healing and signalling for cell differentiation.

## Results

### CSE impairs the migration and viability of AdMSCs

**Figure 1:**
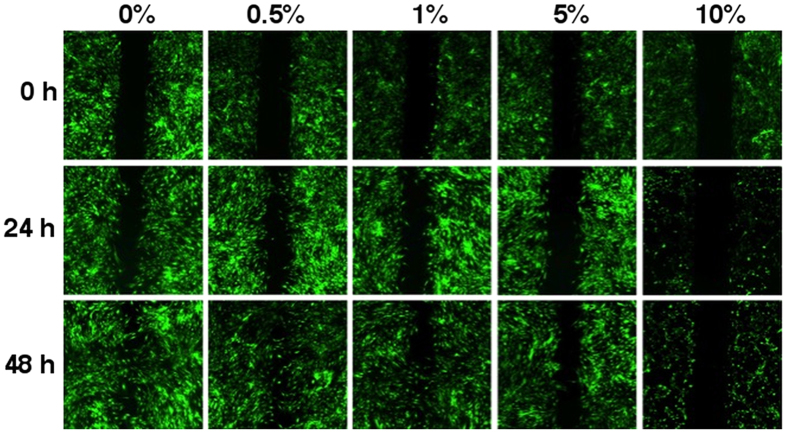
AdMSC migration with CSE exposure. A chamber was used to create a defined open area in between the AdMSCs. The cells were cultured in constant CSE concentrations (0, 0.5, 1, 5, and 10%) after the chamber was removed and were stained with Calcein AM (green) to visualize live cells. After 24 h, migration of the cells was evaluated. Control cells and those exposed to 0.5 and 1% CSE exhibited continual migration through 48 h. The cells exposed to 5% CSE showed very limited migration throughout the experiment and those exposed to 10% CSE scarcely survived.

In order to study cell migration, scratch assays were used where a defined line was created between two confluent areas of AdMSCs using a chamber method (Fig. 1). Cells were exposed to different concentrations of cigarette smoke, ranging from 0.5% to 10%, and their migration into the denuded area was evaluated. A CSE concentration of 0.5% did not show a large difference in migration in comparison to untreated cells. As the concentration of CSE was increased, the migration of the cells visually decreased. At 10% CSE, the cells were unable to migrate and the cell volume significantly decreased.

**Figure 2:**
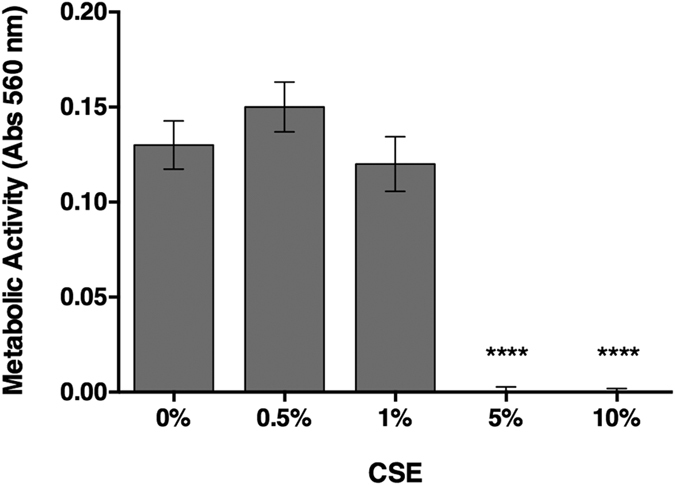
AdMSC survival after CSE exposure. The metabolic activity of the cells was measured 48 h after CSE exposure. The results show that control AdMSCs and those exposed to concentrations up to 1% CSE did not reveal significant differences in metabolic activity. While the cells exposed to CSE concentrations higher than 5% had very limited survival rates. (****p < 0.0001).

A metabolic activity assay was used to determine the viability of the cells exposed to CSE. After 48 h of CSE exposure, at concentrations of 5% and 10%, almost none of the seeded cells were metabolically active (Fig. 2). Exposure of AdMSCs to CSE concentrations under 1% showed no significant differences in cell viability between MSCs exposed to CSE and control cells. Therefore, 0.5% CSE was used for the duration of the study to avoid additional implications and indirect effects.

### CSE affects the secretory profile of AdMSCs

**Figure 3:**
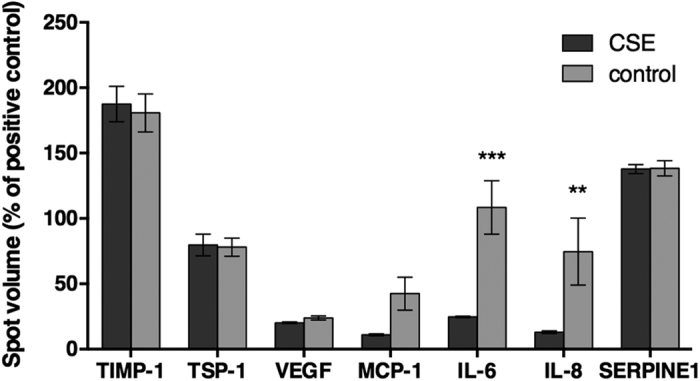
Secretion profile of AdMSCs. AdMSCs were continuously exposed to 0.5% of CSE for 48 h. The conditioned medium was then evaluated for the release of a variety of cytokines and chemokines. Of those measured, angiogenic cytokine IL-6 and chemokine IL-8, important for migration and promoting angiogenesis, showed significant differences. (**p < 0.01, ***p < 0.001).

It has been shown that the expression and release of cytokines and chemokines play an important role in cell signalling and tissue regeneration. Therefore, 36 cytokines and chemokines were simultaneously analysed for differences upon CSE exposure for 48 h. Of the analysed proteins, 7 were highly expressed, including tissue inhibitor of metalloproteinase (TIMP)-1, thrombospondin (TSP)-1, vascular endothelial growth factor (VEGF), monocyte chemotactic protein (MCP)-1, Interleukin factor-6 (IL-6) and −8 (IL-8), and SERPINE1. IL-6 and IL-8 were the only ones to show a significant difference between treatment and control cells with a decrease (*p* < 0.05) in expression in cells exposed to CSE (Fig. 3).

### Differentiation potential of AdMSCs exposed to CSE

After observing that CSE affects key parameters of AdMSCs, such as viability, migration, and the secretory profile, we were interested in studying if CSE could also affect cell differentiation. In this case, the cells were exposed to CSE at the time of differentiation induction, but not before. After the cells had been given supplementation and time enough to differentiate, they were stained for key factors and the gene expression at different time points was analysed. The cells were induced to differentiation into adipogenic, osteogenic, and chondrogenic lineages.

**Figure 4:**
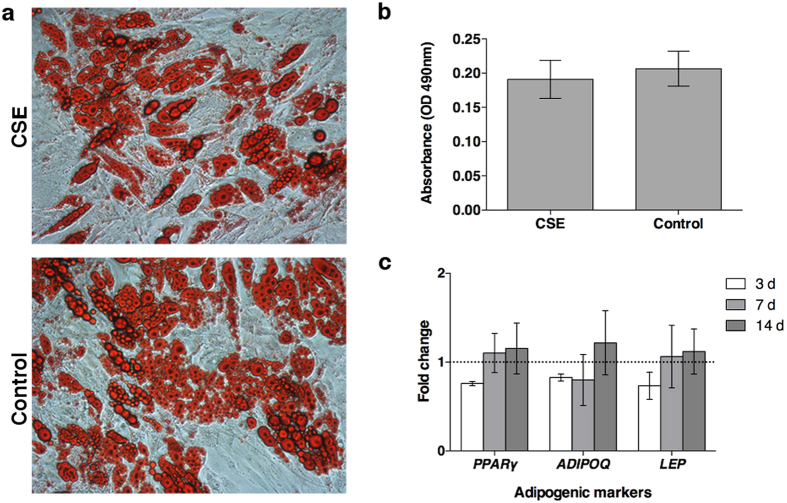
Lipid vacuole formation and gene expression for adipogenic differentiation. AdMSCs were allowed to reach confluency and were then exposed to 0.5% CSE at the moment of induction of adipogenic differentiation with supplemented medium and for 24 h after induction. (**A**) After 21 d in culture, the cells were stained with Oil Red O to detect lipid vacuoles (10× magnification). (**B**) The Oil Red O solution was eluted and the optical density was measured, indicating there were no significant differences in lipid vacuole formation after acute CSE exposure. (**C**) RNA was extracted from the cells and the gene expression of early (*PPARγ*), late (*LEP*), and abundant (*ADIPOQ*) markers was analysed with qPCR, revealing no significant differences after 3 d.

The CSE exposed cells induced to differentiate to the adipogenic lineage did not show significant differences in the adipose specific staining of lipid vacuoles after 21 days or gene expression at 1, 3, 7, or 14 days when compared to controls (Fig. 4 (data from day 1 not included)). Early (*PPARγ*), late (*LEP*), and abundant (*ADIPOQ*) gene markers were used to detect differences over a two-week period but no significant differences arose between the treatment groups.

**Figure 5:**
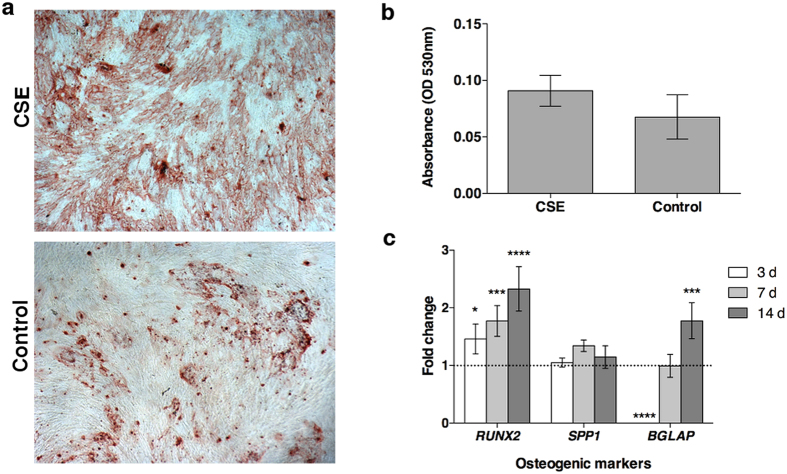
Calcium deposition and gene expression for osteogenic differentiation. AdMSCs were seeded and allowed to attach overnight before being exposed to 0.5% CSE at the time of osteogenic induction. The CSE was allowed to remain on the cells for 24 h. (**A**) After 21 d in culture, the cells were stained for calcium deposition with Alizarin Red S solution (4× magnification). (**B**) The staining solution was removed and the absorbance was read at 530 nm. There were no significant differences detected from the staining after acute CSE exposure. (**C**) To examine the effects on gene expression an early transcription factor (*RUNX2*), an early structural protein (*SPP1*), and a late osteoblastic marker (*BGLAP*) were evaluated. At day 3, the differentiation seems to be slowed in cells exposed to CSE, however expression of late osteoblastic markers was increased after CSE exposure. (*p < 0.05, ***p < 0.001, ****p < 0.0001).

Calcium deposition after 20 days in culture and gene expression was used to assess the differentiation to the osteogenic lineage. While no significant differences were observed in the osteogenic Alizarin Red S staining of the CSE exposed cells, differences at the genetic level were detected (Fig. 5). *RUNX2*, an early transcription factor, showed significantly high (*p* < 0.05) levels of expression compared to control cells indicating the cells are differentiating but seem to remain in an early phase compared to control cells. *SPP1*, a structural protein dependent on the expression of *RUNX2* did not show any significant differences between treatment and control cells. The late osteoblastic marker, *BGLAP*, showed almost no expression at day 3 in CSE exposed cells and had similar expression after 7 days, but after two weeks the expression was double that of the control cells.

**Figure 6:**
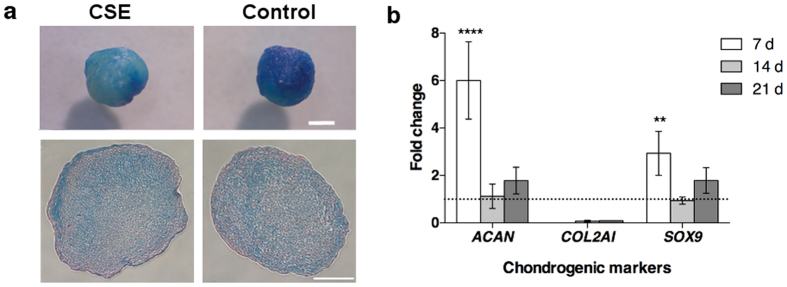
Mucins and gene expression for chondrogenic differentiation. AdMSCs were centrifuged in order to form pellet cultures and induced with chondrogenic supplementation while simultaneously being exposed to 0.5% CSE for 24 h. (A) After 21 d in culture, the pellets were stained with Alcian Blue and sections (5 μm) were further stained with Nuclear Fast Red. A more even stain is observed in the control cell pellets (Scale bar = 1 mm). (B) The gene expression was analysed via a proteoglycan promoter (*ACAN*), structure and strength building proponent (*COL2AI*), and a chondrogenic transcription factor (*SOX9*). While the CSE exposed pellets seem to have a much higher expression of *ACAN* and *SOX9* initially, the levels become comparable at later time points. In addition, based on the lack of *COL2AI*, CSE exposed pellets would indicate a cartilaginous material with poor extracellular matrix and mechanical function. (**p < 0.01, ****p < 0.0001).

Lastly, the chondrogenic differentiation potential of AdMSCs was evaluated upon CSE exposure. Visually, the chondrogenic pellets and sections showed a more uniform Alcian Blue stain with control cells after 21 days (Fig. 6a). Little to no levels of *COL2A1,* the gene that encodes for type-2 collagen in cartilage, was present in cell pellets exposed to CSE. Indicating that the structure and strength of cartilage exposed to CSE may be weakened, as *COL2A1* is necessary for healthy cartilage development and expressed mostly in proliferating chondrocytes. Although, the expression of ACAN, a cartilage specific proteoglycan vital to the extracellular matrix, revealed a very high level of expression at day 7 compared to pellets under control treatments (Fig. 6b). After two weeks the expression was similar from both treatments. *SOX9*, a transcription factor necessary for chondrogenic differentiation, was significantly increased after 7 days of CSE exposure compared to control cell pellets, but did not show any further increase or difference of expression at later time points.

## Discussion

The goal of this study was to examine the consequences of CS on human AdMSCs by means of their migration, viability due to concentration differences, as well as their secretion profile, and differentiation potential under a concentration that was not toxic to the cells alone. The migration and viability of the cells were not significantly affected with concentrations below 1% CSE. When exposed to 5% CSE or higher the cells migration was impaired and they were scarcely metabolically active, presumably due to cell damage. Therefore, the study was carried out with 0.5% CSE where the metabolic activity was comparable to control cells and migration was unaffected (Figs 1 and 2). This concentration was also chosen over 1% CSE to show the effects of a low level of CSE exposure and avoid any possible further detrimental effects. Cell migration and proliferation has been shown to be impaired in periodontal ligament–derived stem cells from smokers^22^. Epithelial cells exposed to CSE revealed increased proliferation and wound repair at low doses^21^. Hematologic effects have been seen in cigarette smokers due to MSC damage by decreasing the amount of cells and proliferative signalling proteins^23^.

After 48 h of incubation with 0.5% CSE, AdMSCs showed a significant downregulation of IL-6 and IL-8. Both of these factors are known to influence angiogenic potential, which would be important in wound healing and fracture repair. In comparison to other studies, our results did not show any significant decrease in VEGF expression. For example, a study with murine models injecting nicotine into dermal wounds found a downregulation in VEGF and minimal change in inflammatory markers, such as IL-6^24^. Another study looking at the effect of nicotine on spinal fusion also revealed a decrease in expression of VEGF with exposure to nicotine^25^. These studies examined the effects of nicotine alone while our study implemented cigarette smoke extract as a whole, including nicotine. While nicotine alone affected the levels of VEGF and IL-6 release, our study did not show an effect on VEGF but revealed a downregulation of IL-6 and IL-8. These differences suggest that the exposure of all of the components in cigarette smoke extract simultaneously, and not just nicotine, have distinct effects on AdMSCs.

SERPINE1, a serine peptidase inhibitor, has exhibited elevated levels in lung cell lines from smokers with emphysema^26^. TSP-1, a regulator of angiogenesis and inflammation, has been shown to be upregulated in the lung fibroblasts of chronic smokers leading to an increased risk of emphysema^27^,^28^. MCP-1, a cytokine which recruits monocytes and phagocytosis, has demonstrated increased levels of expression in cigarette smokers in retinal pigment epithelium^29^. In this study the level of expression was comparable between treatment and control cells for these three factors. The time of exposure in this study was one dose over a 48 h period. This may indicate that long term or chronic exposure has an effect on the protein release. Anti-inflammatory TIMP-1 showed a similar expression in both treatment and control AdMSCs. In humans, TIMP-1 has been shown to have similar release from alveolar macrophages in smokers with normal lung function and non-smokers but elevated in patients with chronic obstructive pulmonary disease^30^.

The adipogenic differentiation showed no significant differences in lipid vacuole formation or gene expression throughout the duration of the study. Adipocytes exposed to CSE trap high molecular weight adiponectin inhibiting its release, which results in an increased likelihood of type 2 diabetes^31^. In the mentioned study, the analysis was taken after differentiation and CSE exposure without recovery time. In our approach, the cells were exposed to CSE at the time of differentiation and it may have been too early for differences in gene expression after one day of induction. On the other hand, the cells may have been able to recover during the 48 h before analysis where they were free of CSE.

Smoking is known to lead to impaired wound healing and fracture repair as well as poor recovery after meniscal surgery^32^,^33^. Interestingly, even though the effects of smoking have been shown to begin diminishing after quitting^34^, nearly a month after low level CSE exposure, effects could still be detected with the cells induced to differentiate into the osteogenic and chondrogenic lineages.

A high expression of *RUNX2*, an early transcription factor for osteoblastic differentiation, may indicate that the cells are able to differentiate but are slower to make the transition when exposed to CSE. The recovery seen in the levelling out of expression of *BGLAP,* a late osteoblastic marker, reveals that the cells are able to recover after a one-week period. Although the expression of *BGLAP* is almost doubled after two weeks in culture indicating excessive osteoblast growth which may compensate for recovery time after injury. In general, smokers have an increased risk of osteoporosis, which in turn increases the risk of bone fractures^35^. The expression of *SPP1,* a structural protein seen early in differentiation, showed similar levels of expression throughout the two weeks, signifying that the structure is not being compromised but the signalling is affected.

As indicated in the osteogenic gene expression, the structure of the chondrogenic pellets seems to have been effected. During differentiation to chondrocytes, *COL2AI* and *ACAN* are typically produced by the cells^36^. *SOX9*, a transcription factor found at various stages of cartilage development, has been shown to enhance expression of aggrecan, a structural protein encoded by *ACAN,* in cartilage-derived cell lines^37^. This also may be the case with differentiated AdMSCs. Overexpression of *ACAN*, a major component of the cartilage extracellular matrix and imperative to chondrogenesis, has been shown to be associated with chondroid accumulation which can occur due to injury^38^. Although *COL2AI* expression has shown elevated expression after one week^39^, this was not the case in our study with AdMSCs exposed to CSE. Almost no expression was present at all indicating that the quality of the pellet exposed to CSE has a low compressive resistance compared to those under control conditions. That *COL2AI* expression lacking in CSE exposed cells indicates a softer cartilage that increases the risk of osteoarthrosis^40^.

The AdMSCs used in this study were taken from healthy non-smoking donors in order to see direct effects of CSE on uncompromised cells. Further investigations examining the differences in cells from donors that smoke may show distinct results. Although the process of passaging and cellular proliferation *in vitro* may remove the effects from a normal or heavy smoker’s cells and those who have already developed CS related diseases may show variation.

Our results describe, for first time, a direct effect of cigarette smoke in the functionality and viability of human AdMSCs *in vitro*. CSE exposure with concentrations higher than 5% drastically impaired the cells. Here we showed that while some recovery is possible with low dose CSE exposure, there are lasting effects on the AdMSCs for cellular signalling with impaired osteogenic and chondrogenic lineages. Further studies should be performed to evaluate healing *in vivo* as well as to study differences between chronic and acute stimulation over time. In addition, the exact mechanism involved that impaired the cellular migration after acute CSE exposure would be advantageous to further understanding how the cells are being inhibited.

## Methods

### Human AdMSC isolation & culture

Human AdMSCs were obtained from lipoaspirates from patients undergoing fat removal surgeries who had given informed consent to participate in the study in the Department of Plastic Surgery and Hand Surgery at the University Hospital rechts der Isar (Munich, Germany). All experimental protocols were approved by the ethical committee of University Hospital rechts der Isar and were carried out in accordance with the approved guidelines. The cells were isolated according to^41^. AdMSCs were cultured in Dulbecco’s modified Eagle’s medium (DMEM; Biochrom, Berlin, Germany) supplemented with 10% Fetal Bovine Serum Gold (FBS; PAA, Pasching, Austria) and 1% antibiotic/antimycotic solution (Capricorn Scientific, Ebsdorfergrund, Germany). Medium was changed two times a week and cells were expanded until use, at passage three. All experiments were carried out singularly with at least three donors (N = 3) in triplicate (n = 3).

### Cigarette smoke extract preparation

Cigarette smoke extract (CSE) was prepared fresh before each experiment and applied to cells within 10 min, according to ISO 3308:2012 conditions^42^. Briefly, a gas-washing bottle with 25 ml DMEM was connected to a peristaltic pump (Watson-Marlow 501, Rotterdam, Netherlands). Three Marlboro red cigarettes, with the filter removed, were smoked for 15 min. Once a minute, for two sec, the valve was closed in order to mimic an inhalation. The resulting extract was then filtered (100 μm mesh; BD Biosciences, San Jose, CA, USA) to remove particulate matter and further filtered (0.22 μm sterile syringe filter; TPP, Trasadingen, Switzerland) to sterilize the extract before being added to the cells at the respective dilution (% v/v).

### AdMSC viability in CSE

To show cellular viability, the metabolic activity was determined using MTT (3-(4,5-Dimethyl-2-thiazolyl)-2,5-diphenyl-2H-tetrazolium bromide) assay (Sigma, Carlsbad, CA). The AdMSCs were seeded in 48 well plates with 5,000 cells per well. Forty-eight hours after the initial seeding, the medium was removed and replaced with medium containing CSE and incubated for an additional 48 h (N = 8, n = 4). MTT reagent was added to each well and incubated for 2 h under standard cell culture conditions. DMSO was used to dissolve the resulting formazan and the optical density was measured with a Mithras LB 940 absorbance reader (Berthold Technologies, Bad Wildbad, Germany) at 560 nm. Blank values from empty wells were then subtracted from sample values.

### CSE exposed AdMSC migration

AdMSCs were seeded in ibidi Culture-Inserts (ibidi GmbH, Martinsried, Germany) with 10,000 cells per chamber in 70 μl of supplemented DMEM. The cells were left in standard cell culture conditions overnight to attach and become confluent. The chambers were then removed and medium containing fresh CSE (0, 0.5, 1, 5, and 10%) was added to the cells. At 0, 24, and 48 h, AdMSCs were stained with Calcein AM (Invitrogen, Carlsbad, CA, USA) and imaged in DMEM with a fluorescent microscope (Zeiss, Göttingen, Germany) to track migration.

### Secretion profile

AdMSCs were plated at a density of 100,000 cells in a T75 cell culture flask (Greiner Bio One, Frickenhausen, Germany). Cells were allowed to attach overnight before experimental conditions were implemented. Control cells only contained supplemented DMEM, while experimental samples had 0.5% CSE. After 48 h at standard cell culture conditions, the supernatants were collected, flash frozen in liquid nitrogen, and stored at −80 °C until the time of analysis. Antibody arrays were performed in order to simultaneously analyse the relative level of 36 different cytokines and chemokines (Human Cytokine Array Panel A, R&D) produced by the cells. Briefly, nitrocellulose membranes spotted with capture antibodies were used to detect the presence of several proteins simultaneously. Membranes were imaged using a Peqlab Fusion FX7 chemiluminescence system (Erlangen, Germany) and the spot intensity was quantified with ImageJ software^43^ using the MicroArray Profile plugin (OptiNav, Inc., Bellevue, WA, USA).

### Differentiation and staining

The abilities of the AdMSCs to differentiate into adipogenic, osteogenic, and chondrogenic lineages was examined when 0.5% CSE was added at the initial point of induction and remained for 24 h. Following this time, the cells were treated under standard differentiation procedures following Lonza induction medium (Walkersville, MO, USA) protocols. Quantitative results were analysed with an unpaired *t*-test.

#### Adipogenic differentiation

Cells were seeded at a density of 21,000/cm^2^ and left to attach for 2–3 d until they reached confluency, upon which time they were stimulated with CSE and adipogenic induction medium (Lonza). The medium changes alternated between induction and maintenance medium following manufacturer’s instructions. After 21 d in culture, Oil Red O (Sigma-Aldrich, St Louis, MO, USA) was used to stain and quantify the resulting lipid droplets. Briefly, the cells were fixed with formalin, rinsed with PBS, and 60% isopropanol was used to eliminate background. The wells were allowed to dry and Oil Red O solution was added for 10 min. The wells were then rinsed with water and imaged. To elute the Oil Red O solution, 100% isopropanol was added for 10 min on an orbital shaker and the optical density of the solution was measured at 490 nm, 0.5 sec.

#### Osteogenic differentiation

Cells were seeded at a density of 3,100/cm^2^ and left to attach overnight before being stimulated with CSE and osteogenic induction medium (Lonza). After 21 d in culture, cells were stained with Alizarin red S (Sigma-Aldrich) to visualize and quantify calcium deposition. Briefly, the cells were fixed with formalin, rinsed with deionized water, and stained with 0.5% Alizarin Red S solution. The cells were rinsed with deionized water and imaged. The quantification of the staining was carried out following the method of^44^ and the absorbance was read at 530 nm.

#### Chondrogenic differentiation

Cells were differentiated as pellet cultures in 15 ml polypropylene conical tubes with 2.5 × 10^5^ per pellet. The pellets were formed by centrifugation and CSE was added at the time of induction with chondrocyte induction medium supplemented with TGF-β3 (Lonza). After 24 h the medium was changed to fresh TGF-β3 supplemented chondrocyte induction medium followed by new medium every 3 d. After 21 d, the pellets were fixed in formalin, and either paraffin embedded and sectioned (5 μm) or left intact and stained with Alcian blue to visualize glycosaminoglycans followed by a Nuclear Fast Red (Sigma-Aldrich) counterstain.

#### Adipogenic and osteogenic RNA isolation

At days 1 (only for the AdMSCs exposed to adipogenic medium), 3, 7, and 14 cells were rinsed with sterile PBS and 350 μl of Buffer RLT (Qiagen, Hilden, Germany) with beta-mercaptoethanol was added to lyse the cells for 5 min. Remaining cells were removed with a cell scraper. The cells were then homogenized in QiaShredders (Qiagen) and RNA was isolated using a Qiagen RNeasy Microkit.

#### Chondrogenic pellet RNA isolation

Pellets were collected in TriZol reagent (Invitrogen, Carlsbad, CA, USA) and shock frozen in liquid nitrogen at days 7, 14, and 21 following the method described in Solchaga, *et al.*^45^. Briefly, the pellets went through various freeze-thaw cycles over four days. After an overnight storage at −80 °C, pellets were broken down with a handheld Ultra-Turrax T10 basic homogenizer (IKA^®^, Staufen, Germany) and further stored at −80 °C. The following day, samples were thawed, chloroform was added, and centrifuged to separate the RNA from the DNA and proteins. After another overnight storage, samples were thawed, washed with ethanol, and allowed to air dry for a short time. RNA pellets were resuspended in 14 μl RNase free water and put on a heat block at 60 °C for 10 min to let the pellet dissolve. The resulting RNA was quantified using a NanoDrop 2000 spectrophotometer (Thermo Scientific, Karlsruhe, Germany).

### Quantitative real time polymerase chain reactions (qPCR)

Complementary DNA (cDNA) was synthesized from the RNA using a QuantiTect Reverse Transcription Kit (Qiagen). To measure the effects of CSE on cells, gene expression was quantified using TaqMan primers (Applied Biosystems, Foster City, CA, USA) for qPCR analysis from cells exposed to CSE and those under standard differentiation conditions. Cells exposed to adipogenic differentiation medium were checked for expression of *PPARγ* (ID: Hs01115513_m1)*, ADIPOQ* (ID: Hs00605917_m1), and *LEP* (ID: Hs00174877_m1). Cells exposed to osteogenic differentiation medium were checked for *RUNX2* (ID: Hs00231692_m1), *SPP1* (ID: Hs00959010_m1), and *BGLAP* (ID: Hs01587814_g1), while chondrogenic expression was assessed by checking for expression of *ACAN* (ID: Hs00153936_m1)*, COL2AI* (ID: Hs_00264051_m1)*, and SOX9* (ID: Hs00165814_m1). Amplification conditions were 50 °C for 2 min, 95 °C for 10 min, 40 cycles at 95 °C for 15 s, and 60 °C for 1 min. Results were normalized to *RPL13* (ID: Hs00744303_s1) transcription level to yield ΔC_t_ and control results to yield ΔΔC_t_. Fold change in expression was calculated using the formula 2^−ΔΔCt^ (Applied Biosystems). Significance was measured using a two-way ANOVA with a Sidak post test.

### Statistical analysis

Results were analysed with GraphPad Prism^®^ version 6.0e for Mac OSX (GraphPad Software, San Diego, CA USA) and are detailed as the mean ± standard deviation. Significant differences were determined by analysis of variance (ANOVA) with a Sidak post-test or an unpaired *t*-test where *p* < 0.05 was considered statistically significant.

## Additional Information

**How to cite this article**: Wahl, E. A. *et al.* Acute stimulation of mesenchymal stem cells with cigarette smoke extract affects their migration, differentiation, and paracrine potential. *Sci. Rep.* **6**, 22957; doi: 10.1038/srep22957 (2016).
